# Analyses of abdominal adiposity and metabolic syndrome as risk factors for respiratory distress in COVID-19

**DOI:** 10.1136/bmjresp-2020-000792

**Published:** 2020-12-16

**Authors:** Cathelijne M van Zelst, Matthijs L Janssen, Nadine Pouw, Erwin Birnie, Manuel Castro Cabezas, Gert-Jan Braunstahl

**Affiliations:** 1 Pulmonology, Franciscus Gasthuis en Vlietland, Rotterdam, Zuid-Holland, The Netherlands; 2 Department of Pulmonology, Erasmus Medical Center, Rotterdam, Zuid-Holland, The Netherlands; 3 Internal Medicine, Franciscus Gasthuis en Vlietland, Rotterdam, Zuid-Holland, The Netherlands; 4 Department of Clinical Chemistry, Franciscus Gasthuis en Vlietland, Rotterdam, Zuid-Holland, The Netherlands; 5 Department of Statistics and Education, Franciscus Gasthuis en Vlietland, Rotterdam, Zuid-Holland, The Netherlands; 6 Department of Genetics, University Medical Centre Groningen, Groningen, Groningen, The Netherlands; 7 Department of Internal Medicine, Franciscus Gasthuis en Vlietland, Rotterdam, Zuid-Holland, The Netherlands

**Keywords:** respiratory infection, respiratory measurement, viral infection

## Abstract

**Background:**

Several characteristics of the metabolic syndrome, such as obesity and hypertension, have emerged as risk factors for a poor clinical outcome in COVID-19. However, most reports lack data on the metabolic syndrome itself. This study investigated prospectively the relationship between respiratory deterioration and the presence of metabolic syndrome or abdominal adiposity in patients with COVID-19.

**Methods:**

A prospective observational cohort study analysing patients with respiratory symptoms who presented at a local emergency department in the Netherlands. The influence of abdominal adiposity—assessed by an increased waist–hip ratio—and metabolic syndrome on respiratory deterioration and the length of hospital stay were analysed with multivariable logistic regressions and Kaplan-Meier analyses.

**Results:**

In total, 166 patients were analysed, of whom 86 (52%) tested positive for COVID-19. The prevalence of metabolic syndrome did not differ between patients with COVID-19 with and without the need for intubation or level of supportive care (37.5% vs 48.4%, p=0.338). In contrast, abdominal adiposity is an independent risk factor for respiratory distress in COVID-19, adjusted for metabolic syndrome, age, gender and BMI (OR 1.11, 95% CI 1.02 to 1.20, p=0.014).

**Conclusion:**

This study shows that abdominal adiposity, and not the presence of metabolic syndrome, is associated with clinical deterioration in COVID-19. This prospective study provides further insight into the risk stratification of patients with COVID-19 based on a simple measurement as the waist and hip circumference.

**Trial registration number:**

NL8580.

**Key question**

Is the metabolic syndrome and/or abdominal adiposity associated with respiratory distress in COVID-19?

**Bottom line**

- In contrast to the metabolic syndrome, it was shown that abdominal adiposity, as measured by an increased waist–hip ratio, is strongly associated with an unfavourable outcome in COVID-19.

**Why read on**

- In an extensive COVID-19 study, the presence of metabolic syndrome and abdominal adiposity measured non-invasively by waist and hip circumference, levels of adipocytokine and duration of hospitalisation were analysed and related to oxygen demand.

## Introduction

Since the outbreak of COVID-19 (SARS-CoV-2) in China in the winter of 2019, the disease has spread rapidly, causing a pandemic. Although the vast majority of patients has only mild upper airway symptoms, a significant proportion of patients suffers from clinically relevant respiratory distress and requires hospitalisation. Several retrospective cohort studies have described the characteristics of admitted patients, thereby providing insight in the subgroups at risk for clinical deterioration. It was observed that, among others, obesity, diabetes and hypertension were prevalent comorbidities in patients hospitalised with COVID-19.^1–3^ The majority of patients suffering from COVID-19 who were admitted to the intensive care unit (ICU) or deceased had at least one of these comorbidities.^4 5^ Moreover, obesity is a significant independent risk factor for respiratory failure in COVID-19,^6^ as observations in 2009 influenza A infection reveal.^7^ An explanation for this observation was suggested to be a prolonged viral excretion in patients with obesity.^8^ Surprisingly, despite a high prevalence of obesity among patients with hypertension and diabetes,^9^ most reports lack data on the outcome of COVID-19 in patients with and without the metabolic syndrome (MetS). Recently published research suggests that intra-abdominal fat depositions (representing abdominal adiposity) on imaging studies are associated with an unfavourable outcome in COVID-19.^10 11^

Visceral fat depositions secrete more inflammatory cytokines, causing an imbalance in the anti-inflammatory and proinflammatory adipokines, thereby altering the immune response. An imbalance in adipokines, resulting in an elevated leptin–adiponectin ratio, is related to increasing insulin resistance.^12^ Overexpression of proinflammatory cytokines, such as leptin and interleukin 6 (IL-6), causes oxidative stress and endothelial dysfunction.^13^ Recent literature showed that the levels of IL-6, which is also partly derived from adipose tissue, are elevated in patients with COVID-19 with obesity and severe respiratory distress admitted to the ICU.^14 15^ Possibly, this might play a role in the described ‘cytokine storm’ in patients with COVID-19, thereby making patients with obesity susceptible to respiratory failure.

It has been recognised that the association between the type of obesity, insulin resistance and COVID-19 should be investigated in detail.^16 17^ In the current study, we investigated the association between MetS, separate criteria of the MetS and severity of disease course in COVID-19 in terms of the required level of respiratory support and the length of hospital stay. We hypothesised that patients with MetS are at risk for an unfavourable course of COVID-19 within 30 days after presentation at the emergency room (ER).

## Methods

### Study design and participants

The MASC study (acronym of Metabolic syndrome And Severity of COVID-19) is a prospective cohort study, conducted from 16 April 2020 until 23 May 2020 at the Franciscus Gasthuis & Vlietland, Rotterdam, the Netherlands. Consecutive patients, aged ≥18 years, presenting with respiratory symptoms or fever suspect for having COVID-19, were assessed for inclusion. The presence of COVID-19 was confirmed by means of PCR performed on patient material obtained by a nasopharyngeal swab or serologic tests. Patients presenting with respiratory symptoms who repeatedly tested negative on COVID-19 PCR but positive for SARS-CoV-2 antibodies were considered positive for COVID-19. Patients with a ‘do not resuscitate/intubate’ order, patients unable to stand upright (due to respiratory distress or pre-existent comorbidities) or patients without measurements of hip and waist circumference were excluded. Patients were followed prospectively for 30 days after presentation. By order of the local research board, only oral consent but no written patient consent was required for performing measurements.

### Outcomes

#### Primary outcome

Unfavourable outcome of disease was defined as being admitted to the hospital with requirement of maximum respiratory support of 3 L/minute (min) supplemental oxygen or more at any time during follow-up, requirement of supplemental oxygen by means of high-flow nasal cannula intubation or admission to the ICU.

Favourable outcome of disease was defined as being discharged from the hospital having required less than 3 L/min oxygen or no admission to the hospital ward at all. Demand for supplemental oxygen was defined as having an oxyhaemoglobin saturation below 94% on room air.

#### Secondary outcomes

Length of stay in hospital: measured in days with a follow-up time of 30 days after referral to the ER.

#### Definitions

The presence of MetS was defined as the presence of any three of the following five traits, modified after the Adult Treatment Panel III criteria^18^:

- Abdominal adiposity, defined as a waist circumference ≥102 cm in men and ≥88 cm in women, measured in the upright position.
- Serum triglycerides ≥1.7 mmol/L or treatment with lipid-lowering drugs.
- Serum high-density lipoprotein cholesterol (HDL-C) <1 mmol/L in men and <1.3 mmol/L in women.
- Drug treatment for elevated blood pressure.
- Non-fasting plasma glucose (>7.8 mmol/L) or drug treatment for elevated blood glucose.

Anthropometric characteristics: abdominal adiposity was assessed by circumferences measurements of waist–hip ratio as defined according to WHO.^19^

In order to provide a biochemical explanation for the influence of abdominal adiposity, IL-6 and adipocytokine (adiponectin and leptin) levels were measured in blood samples that were drawn at ER admission.

### Data collection

On presentation, baseline characteristics, clinical, laboratory and radiological data were collected prospectively following a predefined study protocol. Key data on demographics, baseline comorbidities and presenting clinical parameters were obtained for all included patients. During physical examination at ER presentation, measurements of waist and hip circumference, height and weight were obtained and blood was drawn for routine medical care and cytokine analyses. Detailed data on clinical outcomes, such as supportive care, (duration of) admission, ICU admission and survival were registered at the end of the 30-day follow-up period. Baseline and outcome data were obtained by means of standardised data collection forms.

Laboratory measurements were carried out at the Department of Clinical Chemistry, Franciscus Gasthuis & Vlietland (Rotterdam, the Netherlands) according to standard procedures. IL-6, adiponectin and leptin heparin plasma concentrations were determined using a commercially available ELISA kit (R&D Systems, Minneapolis, Minnesota, USA), according to the manufacturer’s instructions. The minimum detectable dose (MDD) of the ELISA assays was 0.70 pg/mL (IL-6), 0.891 ng/mL (adiponectin) and 7.8 pg/mL (leptin), respectively. Samples with a concentration lower than the MDD were excluded from analyses.

### Patient and public involvement section

Patients were not involved in study design, but the purpose of the study measurements was explained just before measurement. As (abdominal) adiposity and MetS (criteria) are both relevant public health issues, no patients or public involvement was applicable in development of the research questions. Patients were politely invited for participation and offered the option to decline participation without further explanation or the reason why. Patients or public were not involved in design/conduct of the study, choice of outcome measures or recruitment of other patients. Patients/public were not involved in publication/dissemination of the study results.

### Statistical analyses

Baseline data were compared between prespecified subgroups based on the presence of COVID-19, MetS and favourable or unfavourable outcome. Patients in whom data were insufficient to assess the presence of MetS (see definition abovementioned) were excluded from comparisons between patients with and without MetS. Continuous variables were expressed as median with IQR and differences between groups compared with the Mann-Whitney U test (non-normally distributed variables) or unpaired t-test (normally distributed variables); categorical variables were expressed as number with percentages (%) and compared between groups by χ² test. A two-sided p of <0.05 was considered a statistically significant difference.

Univariable and multivariable binary logistic regression analysis were used to study the association between covariates and the course of disease. This was expressed by ORs, 95% CI and p values. Goodness of model fit was based on the model χ^2^ (p value). In additional analyses, each of the five criteria of the MetS, waist–hip ratio and BMI is adjusted for age and gender in multivariable logistic regression models. Variables with p<0.10 in univariable regression were considered as relevant covariates to be included in a final multivariable postadjusted regression model. Based on an a priori scientific understanding that male gender and increasing age are risk factors for an unfavourable disease course in COVID-19, these variables were included in the multivariable regression regardless of the statistical significance in univariable regression. To avoid overestimation, not more than five variables were included in the final multivariable logistic regression. Multicollinearity (correlation between waist–hip ratio and gender) was checked. Potential confounding was evaluated using stratified analysis. A receiver operating characteristic (ROC) curve was used to validate the discriminative ability of the multivariable logistic regression model. A sample size calculation was up front not possible, therefore a post hoc statistical power for the multiple regression was calculated.

The association between MetS and length of stay in the hospital was analysed by means of univariable and multivariable Cox regression. An event was defined as being discharged from hospital alive, within the follow-up period (30 days after hospital admission). Patients who were still admitted and the end of follow-up were censored at day 30. Again, multivariable regression was performed to adjust for possible imbalances between groups. Based on a priori scientific understanding, the same variables as in multivariable logistic regression were included. Covariates that were statistically significant and/or closely associated in univariable analysis were included in the multivariable model. Kaplan-Meier curves were constructed and stratified by presence of MetS or increased waist–hip ratio (according to WHO criteria^19^) and unfavourable outcome. Data were analysed using IBM SPSS Statistics V.26.

## Results

### Descriptive data

**Figure 1:**
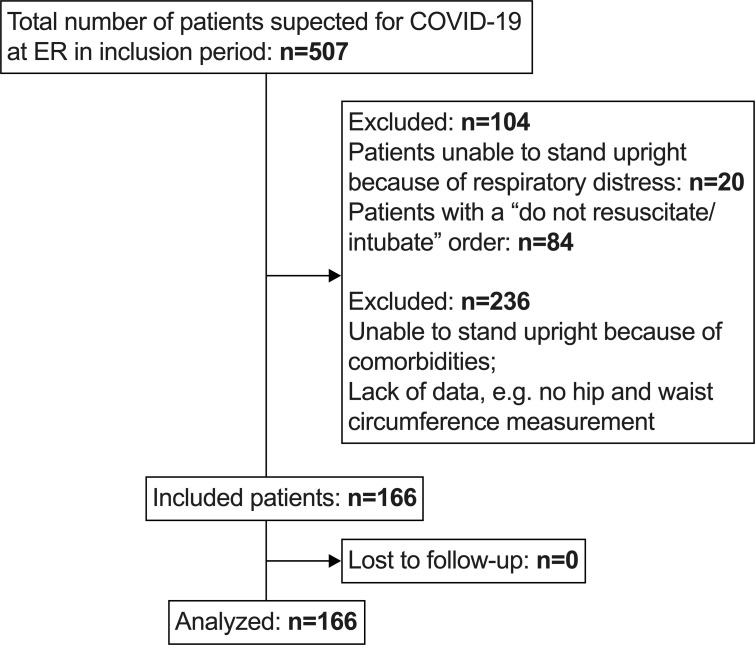
Patient enrolment. ER, emergency room.

From 506 patients presenting at the ER with suspicion of COVID-19, 166 patients were included in the analysis. Patients unable to stand upright because of respiratory distress (n=20), patients with a ‘do not resuscitate/intubate’ order (n=84), patients with a lack of data (no hip and waist circumference measurement) or patients unable to stand upright because of comorbidities (n=236) were excluded for analyses (figure 1). Of the 166 included patients, 86 (52%) tested positive for COVID-19, of whom COVID-19 diagnosis was based on a positive PCR in 80 (93%) and a positive antibody test in six patients (7%). Due to missing data, presence of MetS could not be determined in seven patients, who were therefore excluded from comparisons between favourable and unfavourable groups.

The proportion of patients with MetS was equal in patients positive for COVID-19 and negative for COVID-19 (table 1, 38.7% vs 41.9%, p=0.984). The COVID-19-positive group had a significantly higher median BMI (27.8 vs 26.1 kg/m^2^, p=0.005) and a higher proportion of patients with low HDL-C (84.1% vs 36.8%, p<0.001) compared with the negative group.

**Table 1:** Patient characteristics

	COVID-19 negative (n=79)	COVID-19 positive (n=86)	P value
Male, N (%)	33 (41.8)	40 (46)	0.586
Age, years, median (IQR)	60 (45–67)	56 (47–61.5)	0.267
BMI in kg/m^2^, median (IQR)	26.1 (23.1–29.3)	27.8 (24.7–32.9)	**0.005**
Metabolic syndrome, N (%)	26 (41.9)	33 (38.7)	0.984
Use of antihypertensives, N (%)*	25 (36.2)	22 (25.6)	0.152
High triglycerides, N (%)*	37 (48.7)	29 (34.5)	0.069
Low HDL-C, N (%)*	28 (36.8)	69 (84.1)	**<0.001**
Hyperglycaemia, N (%)*	19 (24.4)	25 (28.7)	0.526
Abdominal adiposity, N (%)*	46 (60.5)	59 (68.6)	0.283
Waist–hip ratio, median (IQR)	0.96 (0.87–1.02)	0.94 (0.88–1.00)	0.492
History of pulmonary disease, N (%)	35 (44.3)	18 (20.7)	**0.001**
History of cardiovascular disease, N (%)	18 (22.8)	18 (20.7)	0.744

Median (IQR Q1–Q3) used in variables with non-normal distribution. Statistically significant p-values are bold.

*Metabolic syndrome criterion. Cut-off values: high triglycerides (>1.7 mmol/L), low HDL-C (<1 mmol/L in male, <1.3 mmol/L in female), hyperglycaemia ≥7.8 mmol/L and/or drug treatment for elevated blood glucose, abdominal adiposity (male ≥102 cm, female ≥88 cm).

BMI, body mass index; HDL-C, high-density lipoprotein cholesterol; N, numbers.

Table 2 shows the clinical parameters of patients with (n=33) and without (n=46) MetS. Number of MetS criteria divided in favourable and unfavourable outcome are shown in online supplemental figure S1. A history of cardiovascular disease was more prevalent among patients with MetS compared with those without MetS (33.3% vs 10.9%, p=0.014). All patients with COVID-19 and MetS and 89% of patients with COVID-19 without MetS were hospitalised (p=0.047). The prevalence of MetS did not differ significantly between patients with a favourable and unfavourable outcome (37.5% vs 48.4%, p=0.338). Among patients with an unfavourable outcome of COVID-19 infection, abdominal adiposity was significantly more prevalent compared with those with a favourable outcome (82.9% vs 58.0%, p=0.015).

10.1136/bmjresp-2020-000792.supp1 Supplementary data

**Table 2:** Characteristics of patients positive for COVID-19

	Without MetS n=46	MetS n=33	P value
Male, N (%)	23 (50.0)	14 (42.4)	0.506
Age, years, median (IQR)	48.5 (34–60.3)	56.0 (45.3–61.8)	0.107
BMI in kg/m^2^, median (IQR)	26.5 (23.8–31.4)	30.4 (27.2–35.8)	**0.004**
MetS, N (%)	–	–	–
Use of antihypertensives, N (%)*	4 (8.7)	17 (51.5)	–
High triglycerides, N (%)*	2 (4.3)	24 (72.7)	–
Low HDL-C, N (%)*	36 (87.3)	33 (100)	–
Hyperglycaemia, N (%)*	2 (4.3)	19 (57.6)	–
Abdominal adiposity, N (%)*	24 (52.2)	29 (87.9)	–
Waist–hip ratio, median (IQR)	0.93 (0.84–0.98)	0.95 (0.90–1.02)	**0.034**
History of pulmonary disease, N (%)	10 (21.7)	6 (18.2)	0.698
History of cardiovascular disease, N (%)	5 (10.9)	11 (33.3)	**0.014**
Use of antibiotics, N (%)	19 (41.3)	10 (30.3)	0.274
Deceased (30-day follow-up), N (%)	0 (0)	0 (0)	–
Discharged from ER without hospitalisation, N (%)	5 (11)	0 (0)	**0.047**
Hospitalisation, N (%)	41 (89)	33 (100)	
Respiratory support†			
Intubation, N (%)	7 (15)	6 (18)	0.989
High-flow nasal cannula, N (%)	4 (9)	3 (9)	
Supplemental oxygen 1–6 L/min, N (%)	24 (52)	21 (64)	
6	–	–	0.111
5	1 (2)	2 (6)	
4	0	3 (9)	
3	4 (9)	1 (3)	
2	9 (20)	11 (33)	
1	10 (22)	3 (9)	
Hospitalised, without oxygen supply, N (%)	6 (13)	4 (12)	
Unfavourable course of disease, N (%)	16 (35)	15 (45)	0.338
Readmission ER related to COVID-19, N (%)	7 (15)	4 (12)	0.658

Sufficient data to diagnose or exclude MetS were missing in seven patients. Median (IQR Q1–Q3) used in variables with non-normal distribution. Statistically significant p-values are bold.

*MetS criterion. Cut-off values: high triglycerides (>1.7 mmol/L), low HDL-C (<1 mmol/L in male, <1.3 mmol/L in female), hyperglycaemia ≥7.8 mmol/L and/or drug treatment for elevated blood glucose, abdominal adiposity (male ≥102 cm, female ≥88 cm).

†Respiratory support was defined as maximum supplemental oxygen at any given moment during hospitalisation.

BMI, body mass index; ER, emergency room; HDL-C, high-density lipoprotein cholesterol; MetS, metabolic syndrome; N, numbers.

### Predictive model for unfavourable outcome

Table 3 shows the results from univariable and multivariable postadjusted logistic regression, analysing the odds of an unfavourable outcome in COVID-19. MetS and all the separate criteria of MetS were also analysed in additional logistic regressions, adjusted for age and gender, showing significant impact on the outcome for both abdominal adiposity and also for BMI (online supplemental table S1). The final multivariable model shows that the waist–hip ratio (OR 1.11, 95% CI 1.02 to 1.20, p=0.014) and BMI (OR 1.11, 95% CI 1.00 to 1.23, p=0.043) were significantly associated with an increased risk for an unfavourable outcome of COVID-19, when adjusted for MetS, age and gender. MetS was not significantly related to an unfavourable outcome in univariable and multivariable logistic regressions. Online supplemental figure 2 shows that the area under the ROC curve was 0.771 (95% CI: 0.664 to 0.878) and post hoc statistical power analysis for this model is 0.85.

**Table 3:** Univariable and multivariable postadjusted logistic regression analyses: association between patient characteristics and severity of COVID-19

Covariate	Univariable logistic regression		Final multivariable postadjusted logistic regression model	
	OR (95% CI)	P value	OR (95% CI)	P value
Constant	–	–	0.000	**0.001**
Male gender	2.28 (0.95 to 5.48)	0.064	0.90 (0.23 to 3.56)	0.884
Age	1.02 (0.98 to 1.06)	0.288	1.01 (0.96 to 1.06)	0.684
Metabolic syndrome	1.56 (0.63 to 3.90)	0.339	0.70 (0.21 to 2.19)	0.506
Use of antihypertensives*	0.58 (0.21 to 1.63)	0.303	–	–
Hypertriglyceridemia*	1.13 (0.45 to 2.83)	0.802	–	–
Low HDL-C*	2.63 (0.67 to 10.42)	0.168	–	–
Hyperglycaemia*	1.79 (0.70 to 4.58)	0.225	–	–
Abdominal adiposity*	3.50 (1.23 to 9.93)	**0.019**	–	–
Waist–hip ratio	1.11 (1.05 to 1.18)	**0.001**	1.11 (1.02 to 1.20)	**0.014**
BMI	1.11 (1.02 to 1.21)	**0.016**	1.11 (1.00 to 1.23)	**0.043**
Post hoc power	–		0.85	

*Metabolic syndrome criterion. Cut-off values: high triglycerides (>1.7 mmol/L), low HDL-C (<1 mmol/L in male, <1.3 mmol/L in female), hyperglycaemia ≥7.8 mmol/L and/or drug treatment for elevated blood glucose, abdominal adiposity (male ≥102 cm, female ≥88 cm). Statistically significant p-values are bold.

BMI, body mass index; HDL-C, high-density lipoprotein cholesterol.

### Length of hospital stay

**Figure 2:**
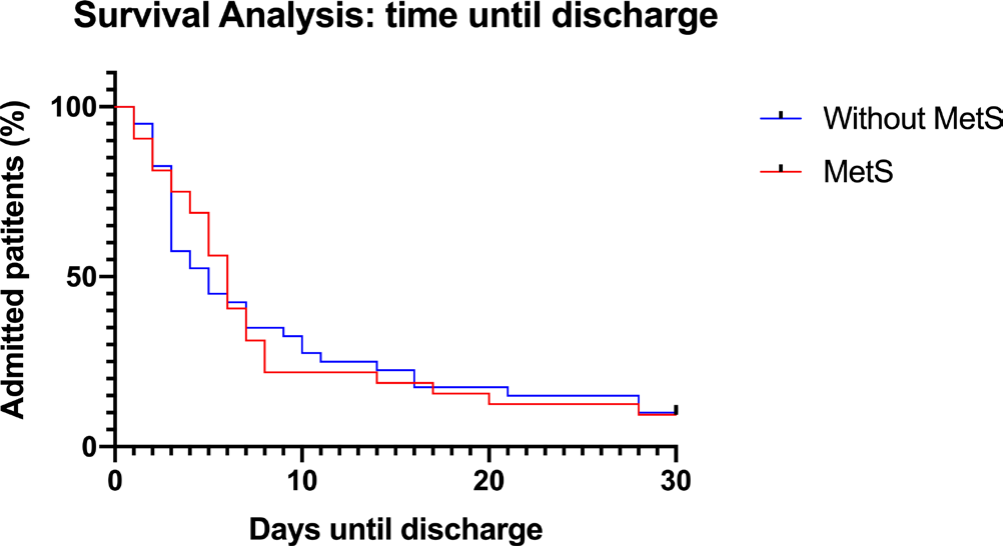
Survival analysis displaying time until discharge in patients with COVID-19 compared between patients with and without metabolic syndrome (MetS). Median time until discharge was 6 days in the MetS group (IQR 3–8) and 5 days in the group without MetS [(IQR 3–11). Log-rank test p value=0.921. Patients who were still admitted and the end of the follow-up period were censored at day 30. No patients died during follow-up.

**Figure 3:**
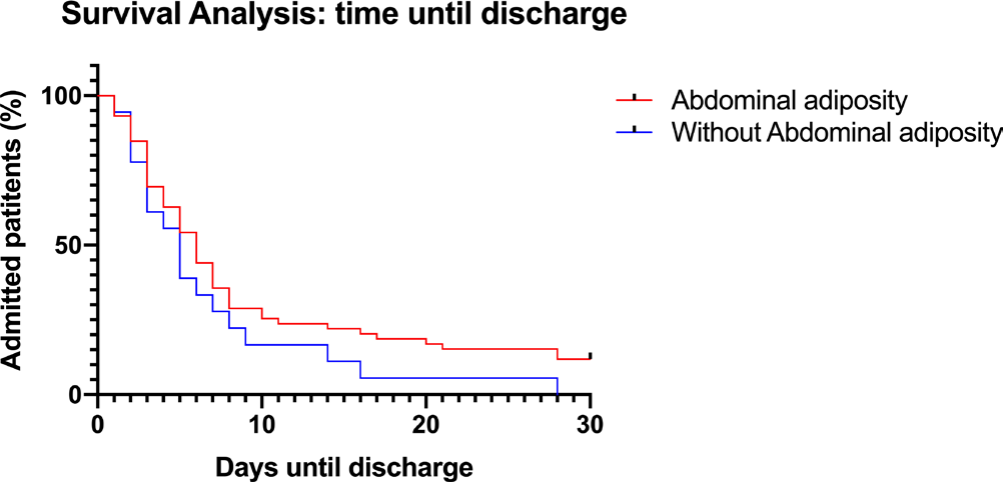
Survival analysis displaying time until discharge in patients with COVID-19, compared between patients with and without abdominal adiposity. Abdominal adiposity is defined according to WHO criteria male >0.9 and female >0.85 based on WHR. Patients who were still admitted and the end of the follow-up period were censored at day 30. Median time until discharge was 4 days in the group without abdominal adiposity (IQR 2–8) and 6 days in the group with abdominal adiposity (IQR 3–11). Log-rank test p value=0.129. WHR, waist–hip ratio.

No patients died during the 30-day follow-up period (table 2). Figure 2 shows duration of hospitalisation for patients positive for COVID-19, with and without MetS. The median (IQR) time until discharge was 6 (3–8) days in the MetS group and 5 (3–11) days in the group without MetS (log-rank test p=0.921). Figure 3 represents the Kaplan-Meier curves of patients with COVID-19 with and without abdominal adiposity based on waist–hip ratio. Median time until discharge was 4 days in the group without abdominal adiposity (IQR 2–8) and 6 days in the group with abdominal adiposity (IQR 3–11). There was no statistically significant difference between the curves (log-rank test p=0.129).

Table 4 shows the univariable and multivariable association between patient characteristics and length of stay using Cox regression. In univariable Cox regression, MetS was not significantly related to length of hospital stay (HR=0.98, 95% CI 0.80 to 2.22, p=0.23). Waist–hip ratio indicated a prolonged length of hospital stay in the univariable Cox regression model (HR=0.98, 95% CI 0.95 to 0.99, p=0.04). However, it did not show a statistically significant effect on length of stay, when adjusted for age and gender (p=0.29).

**Table 4:** Univariable and multivariable Cox regression: association between patient characteristics and time until discharge

Covariate	Univariable Cox regression		Multivariable Cox regression	
	HR (95% CI)	P value	HR (95% CI)	P value
Male gender	0.76 (0.48 to 1.22)	0.25	0.86 (0.45 to 1.61)	0.63
Age (years)	0.99 (0.97 to 1.01)	0.48	0.99 (0.97 to 1.01)	0.68
Metabolic syndrome	0.98 (0.60 to 1.16)	0.93	1.22 (0.69 to 2.15)	0.50
Use of antihypertensives*	1.32 (0.80 to 2.22)	0.23	–	–
Hypertriglyceridemia*	1.16 (0.71 to 1.89)	0.55	–	–
Low HDL-C*	0.80 (0.40 to 1.61)	0.53	–	–
Hyperglycaemic*	0.87 (0.52 to 1.45)	0.59	–	–
Abdominal adiposity*	0.77 (0.47 to 1.27)	0.30	–	–
Waist–hip ratio	0.98 (0.95 to 0.99)	**0.04**	0.98 (0.95 to 1.02)	0.29
BMI	0.97 (0.93 to 1.01)	0.12	0.97 (0.92 to 1.01)	0.12

HR <1 related to prolonged length of stay, HR >1 shortened length of stay. −2Log Likelihood 499.14, χ^2^=7.24, p=0.203. Statistically significant p-values are bold.

*Metabolic syndrome criterion. Cut-off values: high triglycerides (>1.7 mmol/L), low HDL-C (<1 mmol/L in male, <1.3 mmol/L in female), hyperglycaemia ≥7.8 mmol/L and/or drug treatment for elevated blood glucose, abdominal adiposity (male ≥102 cm, female ≥88 cm).

BMI, body mass index; HDL-C, high-density lipoprotein cholesterol.

### Cytokine measurement

**Figure 4:**
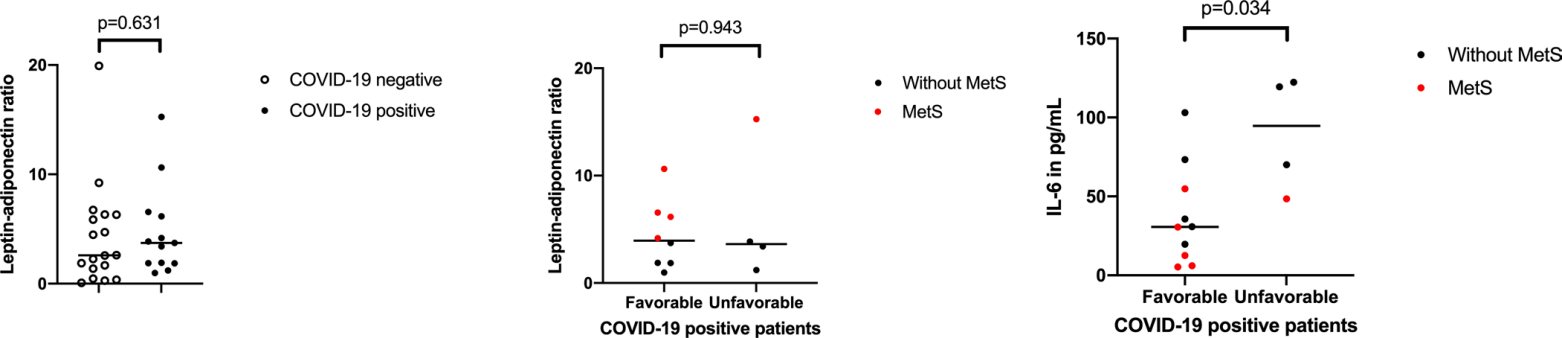
Distribution and comparison of leptin–adiponectin ratio between patients positive for COVID-19 and negative for COVID-19 and patients positive for COVID-19 divided in favourable and unfavourable outcome. IL-6, interleukin 6; MetS, metabolic syndrome.

In a subset of patients, the levels of proinflammatory (IL-6, leptin) and anti-inflammatory cytokines (adiponectin) were measured at ER admission and compared between groups (table 5, figure 4). Plasma samples for cytokine measurement were available from 29 included patients (12 COVID-19-positive and 17 COVID-19-negative). Baseline characteristics were comparable between these two groups table 5Comparison between patients with COVID-19 with and without MetS showed that IL-6 was lower in patients with COVID-19 with MetS (median 70.01 vs 30.60 pg/mL, p=0.028). IL-6 was higher in patients with COVID-19 with an unfavourable outcome than in those without (median 94.7 vs 30.7, p=0.034). The leptin–adiponectin ratio was higher among patients with COVID-19 with MetS compared with those without MetS (ratio 6.6 vs 1.9, p=0.003, table 5. This was not observed when comparing the ratio between patients with an unfavourable outcome to patients with a favourable outcome (p=0.943).

**Table 5:** Cytokine measurements

	COVID-19 negative (n=17)	COVID-19 positive (n=12)	P value	Without MetS (n=7)	MetS (n=5)	P value
**Age in years, median (IQR**)	59.0 (47.30–65.8)	51.5 (40.3–61.0)	0.97	46.00 (40.00–61.00)	54.00 (41.50–70.00)	0.876
**BMI in kg/m^2^, median (IQR**)	27.2 (25.4–29.9)	28.68 (24.28–31.83)	0.507	27.04 (23.51–30.47)	31.86 (25.77–38.98)	0.149
**Waist–hip ratio, median (IQR**)	0.96 (0.89–1.03)	0.95 (0.89–1.01)	0.632	0.95 (0.76–0.99)	0.95 (0.90–1.03)	0.755
**MetS, N (%**)	7 (41.0)	5 (41.7)	0.979	–	–	–
**Leptin (pg/mL), median (IQR**)	20 034.4 (3229.7–30 026.6)	20 870.3 (7593.0–37 178.9)	0.235	20 581.3 (6878.1–29 815.6)	35 643.8 (13 675.0–63 581.3)	0.108
**Adiponectin (ng/mL), median (IQR**)	4467.5 (2834.0–9645.5)	5663.9 (3679.6–8498.3)	0.677	7671.7 (5148.2–9766.0)	3687.4 (2392.9–6233.2)	0.106
**Leptin–adiponectin ratio, median (IQR**)	2.6 (0.9–6.3)	3.8 (1.9–6.5)	0.631	1.9 (1.2–3.7)	6.6 (5.2–12.9)	**0.003**
**IL-6 (pg/mL), median (IQR**)	<MDD	42.08 (22.44–94.81)	–	70.01 (30.8–119.5)	30.6 (9.3–51.7)	**0.028**

Measurements of IL-6, leptin and adiponectin in a subgroup of patients. Several relevant clinical parameters, such as BMI, waist–hip ratio and presence of MetS are shown. Median (IQR Q1–Q3) used in variables with non-normal distribution. Statistically significant p-values are bold.

BMI, body mass index; IL-6, interleukin 6; MDD, minimum detectable dose; MetS, metabolic syndrome; N, numbers.

## Discussion

This study is the first to investigate the influence of abdominal adiposity assessed by the waist–hip circumference, a non-invasive measurement, on COVID-19 outcome. Abdominal adiposity was significantly associated with a more severe course of COVID-19, adjusted for age, gender and BMI. Similar observations are made in recently published studies, analysing the amount of intra-abdominal (eg. visceral) fat depositions in patients with COVID-19.^10 11^ Agreeing with the present study, these investigations show that abdominal adiposity on CT imaging is related to respiratory failure in COVID-19. However, our results are based on measurement of the waist–hip circumference, which is compared with abdominal CT scanning, a clinically more feasible and non-invasive method to assess abdominal adiposity. Overweight (>50%) and obesity (15.9%) are highly prevalent in the general Dutch population.^20^ Patients presenting with respiratory symptoms at our centre who tested positive for COVID-19 had a higher BMI compared with those without COVID-19. Given that (abdominal) adiposity is a treatable risk factor, in contrast to other risk factors such as age and gender, our results underline the relevance of this topic in COVID-19 research. In accordance with recent literature, the present study also describes a relationship between BMI and respiratory failure in COVID-19. But, according to our study, abdominal adiposity seems to be a more important risk factor for an unfavourable outcome in COVID-19. This is in line with earlier reports suggesting that abdominal adiposity is a strong predictor for respiratory deterioration in COVID-19 and in conditions other than COVID-19.^10 11 21–23^

The association between MetS and the severity of COVID-19 infection was investigated in this study, which had never been done before. It was observed that all patients with COVID-19 who fulfilled the criteria for MetS were admitted to the hospital ward, whereas none of the patients without the MetS was admitted. However, the presented results do not support a relationship between MetS and an impaired clinical outcome or duration of admission by COVID-19.

This study also investigated the influence of MetS and abdominal adiposity on the hospital length of stay in patients with COVID-19. Kaplan-Meier analyses comparing patients with COVID-19 with or without MetS showed no significant differences between the groups. The Kaplan-Meier curve comparing patients with and without abdominal adiposity showed a trend towards a longer duration of hospitalisation in patients with abdominal adiposity, agreeing with the finding that abdominal adiposity promotes a more severe course of disease. In univariable analysis, abdominal adiposity was associated with a longer duration of hospitalisation in COVID-19. However, in multivariable Cox regression, there were no clinical factors associated with a prolonged duration of admission.

Several mechanisms may explain the observed relation between abdominal adiposity and respiratory failure. First, visceral fat deposits, which are increased in abdominal adiposity, act as a reservoir for viral load promoting an inflammatory response.^23 24^ Augmented visceral fat volume is also associated with an impaired viral shedding.^8^ Second, an imbalance in proinflammatory and anti-inflammatory adipokines can cause increased respiratory distress. Levels of IL-6, leptin and adiponectin were determined at baseline and compared between groups, in order to test the hypothesis that proinflammatory adipokines are higher in patients with COVID-19 with MetS compared with those without MetS. The presented results, although derived from a small sample size, support the idea that the leptin–adiponectin ratio was elevated in patients with COVID-19 with MetS. No association between the leptin–adiponectin ratio on outcome in COVID-19 could be demonstrated. In addition, higher IL-6 levels were related to an unfavourable outcome. But, IL-6 was elevated in patients with COVID-19 irrespective of the presence of MetS. This might implicate that IL-6 levels in low-grade inflammation are negligible in MetS compared with the levels measured during COVID-19 infection. Earlier studies already showed that IL-6 is elevated in (severe) COVID-19 and is an independent predictor for the need of mechanical ventilation in patients with COVID-19.^14 25–27^

An alternative explanation for the relation between abdominal adiposity and respiratory failure, which was not further investigated in this study, is that increased intra-abdominal pressure due to local fat deposition may lead to mechanical obstruction and impaired ventilation of the lower lung regions. In theory, the large waist circumference may increase small airway resistance and reduce the functional residual capacity (FRC), thereby decreasing lung compliance and causing compression atelectasis.^28^ This has been demonstrated to be an important mechanism of action in other pulmonary conditions, such as asthma.^29^

In contrast to earlier large studies, hypertension or a history of cardiovascular disease, which are common comorbidities among COVID-19 and well-known risk factors for clinical deterioration,^1 4 6 30^ were not associated with severe respiratory failure in our cohort. Newly discovered hypertension at the ER was not included as a criterion of MetS, due to the influence of anxiety and stress caused by COVID-19 or hospitalisation. Possibly, the use of this definition may have caused a small proportion of patients with hypertension among patients with COVID-19 in this study, explaining the discrepancy with previous reports.

The present study has a few limitations. First, the cut-off value for respiratory distress used in our study was a requirement of supplemental oxygen ≥3 L/min. The rationale for this cut-off point was based on clinical experience in our centre. It was observed that the clinical condition in patients with COVID-19 often deteriorated soon after admission and that oxygen demand quickly increased in patients who required oxygen at ≥3 L/min. A cut-off value of ≥3 L/min to define respiratory distress was also used in a study by Demoule *et al*.^31^ In this analysis, 257 out of 397 patients required intubation, thereby underlining the rationale for the applied cut-off value. Second, the sample size is limited, foremost in the measurement of cytokines, and documentation of clinical parameters is incomplete in some cases. However, post hoc analysis revealed a power of 0.85 for the presented model, meaning acceptable power. Finally, some bias in measurement of the waist–hip circumference could not be avoided, as they are supposed to be measured in upright position. Therefore, patients who were unable to stand at admission due to respiratory distress had to be excluded from analysis, thereby introducing selection bias. However, given the small number of patients to whom this applied (20 out of 506), the influence on the results is expected to be limited.

## Conclusion

In the present study, MetS did not appear to be of influence on the clinical outcome in COVID-19. However, the results show a clear association between respiratory deterioration in COVID-19 and abdominal adiposity, assessed by the easily measurable waist–hip circumference. As abdominal adiposity is prevalent worldwide,^32^ the influence on clinical outcome in COVID-19 is equally, if not more, important than the effect of BMI. Further research with bigger sample size, extensive determination of bioinflammatory markers and lung function data (ie, FRC) could help to explain the underlying mechanisms responsible for the association between abdominal adiposity and respiratory distress in COVID-19.
