# Supplementary material for: Analyses of abdominal adiposity and metabolic syndrome as risk factors for respiratory distress in COVID-19
Source: BMJ Open Respir Res. 2020 Dec 16;7(1):e000792. doi: 10.1136/bmjresp-2020-000792 (PMC7745457; doi:10.1136/bmjresp-2020-000792)

Supplemental data

Figure S1. Number of Metabolic Syndrome criteria and severity of COVID-19

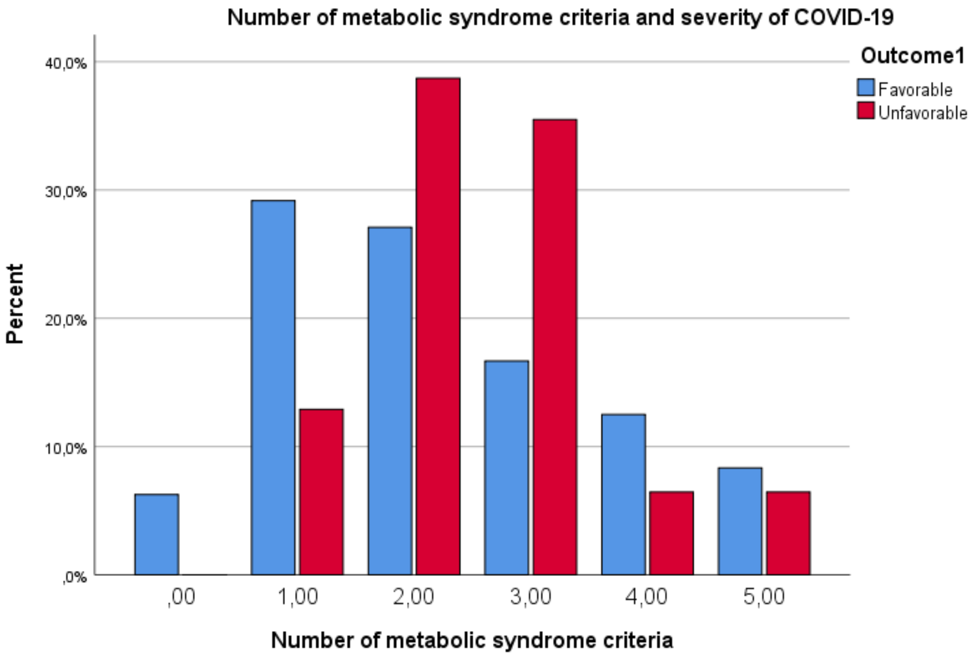

Criteria of Metabolic Syndrome: 1) Use of antihypertensiva, 2) Triglycerides >1.7 mmol/L and/ or statin use, 3) HDL-C <1 mmol/L in male or <1.3mmol/L in female, 4) Hyperglycaemia ≥7.8 and/ or use of medication for glycemic control 5) Abdominal adiposity in male ≥ 102 cm or female ≥ 88cm

Table S1. Multivariable post-adjusted logistic regression models, adjusted for age and gender

	**Multivariable post-adjusted logistic regression models.** All variables are adjusted for age and gender	
**Covariate**	**OR (95%CI)**	**P-value**
**Metabolic Syndrome**	1.64 (0.64-4.25)	0.306
**Use of antihypertensives***	0.42 (0.13-1.36)	0.147
**Hypertriglyceridemia***	0.89 (0.34-2.35)	0.809
**Low HDL-C ***	2.46 (0.60-10.06)	0.211
**Hyperglycaemia***	2.13 (0.77-5.94)	0.148
**Abdominal adiposity***	5.19 (1.61-16.75)	**0.006**
**Waist-hip ratio**	1.13 (1.05-1.22)	**0.001**
**BMI**	1.14 (1.04-1.25)	**0.006**

OR: Odds Ratio, CI: Confidence Interval, BMI: body mass index, HDL-C: High Density Lipoprotein cholesterol. Cutoff values: High Triglycerides (>1.7 mmol/L), Low HDL-C (<1 mmol/L in male, <1.3mmol/L in female), Hyperglycaemia ≥7.8 mmol/L and/ or drug treatment for elevated blood glucose, Abdominal adiposity (male ≥ 102 cm, female ≥ 88cm).

Figure S2. ROC-curve of multivariable post-adjusted regression analysis.

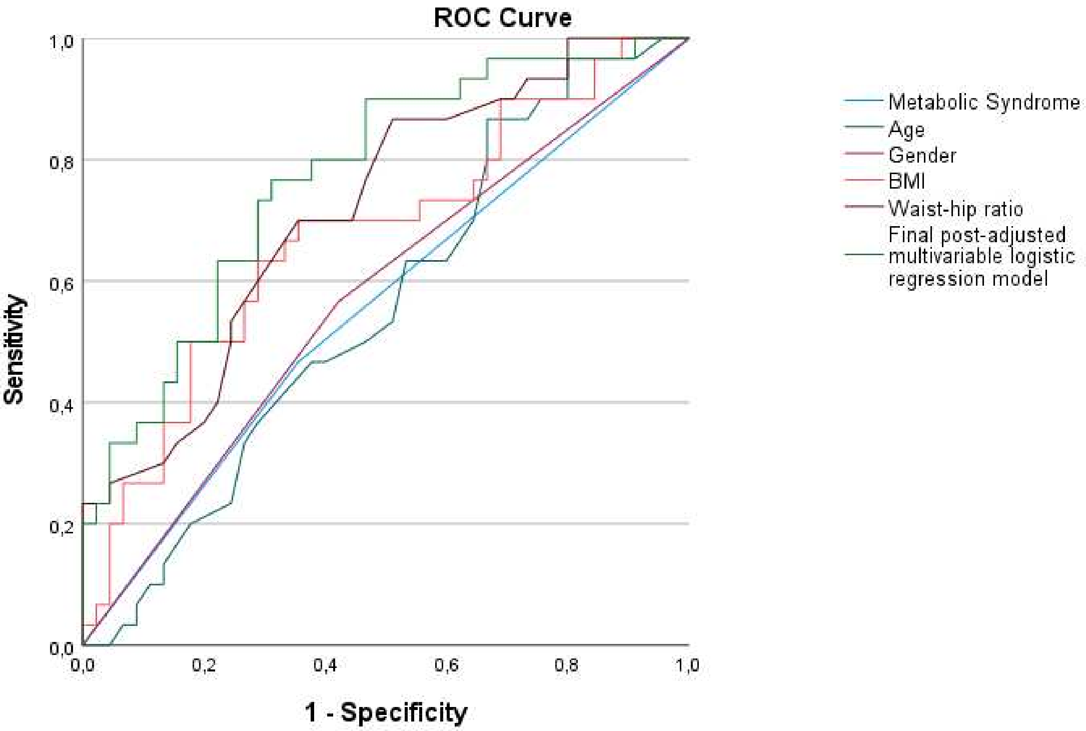

Supplement: Supplementary data [file bmjresp-2020-000792supp001.pdf]
